# Impact of long-lasting moderate-intensity stage cycling event on cardiac function in young female athletes: A case study

**DOI:** 10.1371/journal.pone.0275332

**Published:** 2022-10-04

**Authors:** Solène Le Douairon Lahaye, Gaëlle Kervio, Vincent Menard, Anna Barrero, Thibault Lachard, Guy Carrault, David Matelot, François Carré, Frédéric Schnell

**Affiliations:** 1 M2S Laboratory, ENS Rennes, University of Rennes 2, Rennes, France; 2 CIC-CIT INSERM 1414, Rennes, France; 3 Department of Sports Medicine, University Hospital of Rennes, Rennes, France; 4 INSERM, LTSI-UMR1099, University of Rennes 1, Rennes, France; University of Bourgogne France Comté, FRANCE

## Abstract

**Purpose:**

Effects of intense and/or prolonged exercise have been studied extensively in male athletes. Nevertheless, data are scare on the effect of long duration events on cardiac function in female athletes. Our aim was to investigate the effect of a long-lasting moderate-intensity stage cycling event on cardiac function of young female athletes.

**Methods:**

Seven well-trained female cyclists were included. They completed a cycling event of 3529 km on 23 days. All underwent an echocardiography on 6 time-points (baseline and at the arrival of day (D) 3, 7, 12, 13 and 23). Cardiac function was assessed by conventional echocardiography, tissue Doppler imaging and speckle tracking techniques. Daily exercise load was determined by heart rate (HR), power output and rate of perceived exertion data (RPE, Borg scale).

**Results:**

All stages were mainly done at moderate intensity (average HR: 65% of maximal, average aerobic power output: 36% of maximal, average RPE: 4). Resting HR measured at the time of echocardiography did not vary during the event. Resting cardiac dimensions did not significantly change during the 23 days of cycling. No significant modification of cardiac function, whatever the studied cavity, were observed all along the event.

**Conclusion:**

The results suggest that, in the context of our case study, the long-lasting moderate-intensity stage cycling event was not associated with cardiac function alteration. Nevertheless, we must be careful in interpreting them due to the limits of an underpowered study.

## Introduction

Acute prolonged intense exercise provides a substantial challenge to the cardiovascular system and can induce an exercise-induced cardiac fatigue (EICF) [1–5]. EICF could be evaluated with cardiac biomarkers or imaging methods, especially echocardiography. Numerous studies, conducted mainly in male athletes, observe an alteration in cardiac biomarkers and/or systolic and diastolic functional indicators after acute prolonged intense exercise [6–12]. Echocardiographically, the EICF seems to adopt a cascade: from alteration of speckle tracking derived parameters of diastolic function to an alteration of the conventional markers of systolic function [13]. Nevertheless, in the great majority of cases, this EICF was transient. Indeed, cardiac function and biomarkers returned to normal after 24–48 hours of recovery [6, 7, 14, 15].

However, in the case of multi-day prolonged exercise, athletes usually benefit from short (< 24 hours) recovery periods between stages. Thus, it makes sense to think that the physiological stress placed on the myocardium during repeated and prolonged efforts may result in a cumulative decrement in cardiac function, subsequently delaying the recovery process. Surprisingly, this cumulative cardiac fatigue was not found in most of studies that investigated the impact of multi-day prolonged exercise on cardiac function [16–18].

These studies included essentially male or mixed population athletes. However, females are increasingly participating in these multi-day extended events with some of them exclusively reserved for them (*e*.*g* Raid amazones, Laponie Trophy, Corsica Raid Femina…). Due to different motivations from those of male athletes, the vast majority of them perform these sport events at a moderate intensity, looking for happiness, social needs and physical fitness rather than performance [19, 20].

To our knowledge, the cardiovascular response of female to this kind of physical exercise is less documented [21, 22]. In a previous study on well-trained female cyclists, we have demonstrated that during a multistage event, heart rate (HR) and heart rate variability (HRV) parameters evolved along the event in correlation with the daily physical activity [22]. Nevertheless, the effect of this type of events on specific cardiac function parameters in female athletes remains unknown. Therefore, the aim of the present case study was to study the impact of a long-lasting moderate-intensity stage cycling event on cardiac function of well-trained young female cyclists using conventional and two-dimensional speckle-tracking echocardiographic markers.

## Methods

**Fig 1:**
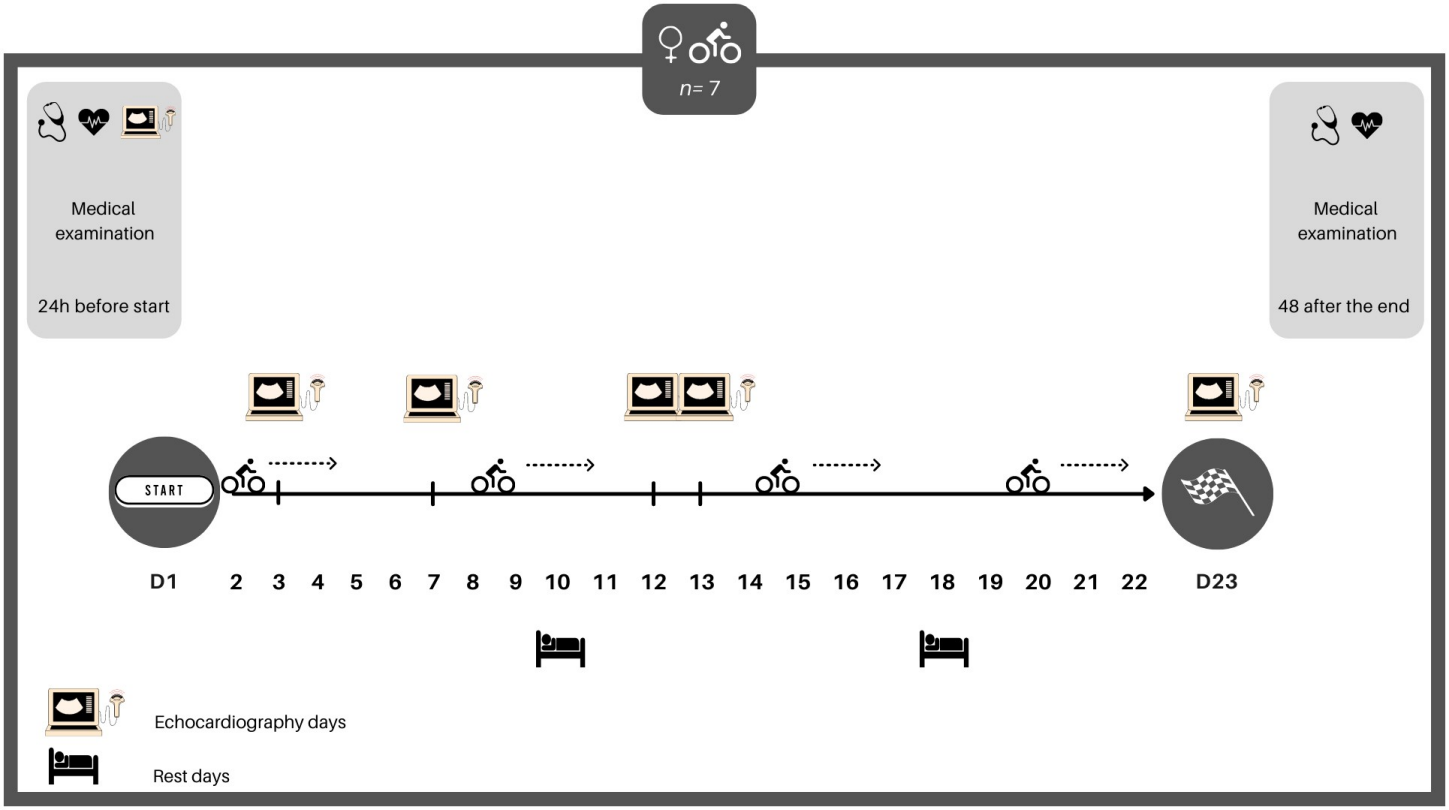
Experimental design.

The experimental design of the case study is presented in Fig 1.

### Population

As in our previous study [22], we included all the members of the sport project “*Donnons des elles au vélo J-1*” (2016 edition) *i*.*e* seven healthy and well-trained (regional or national level) female cyclists coming from 3 countries (Belgium, France, Ukraine). This project sport aims to promote women’s cycling. All subjects were non-smokers, healthy with no history of cardiac disease and without medication. Due to the ecological context of the research project (integrated to sport project), the menstrual cycle for each subject was different at the beginning of the event and thus not controlled. Cyclists reported not taking the pill for at least 6 months. All participants gave their informed consent to participate in this study, which received the approval of the Rennes university hospital ethics committee (Number 2013-A01524-41) and was conducted in accordance with the Declaration of Helsinki.

### Pre- and post-participation medical evaluation

All athletes had a medical examination before and 48h after the end of the cycling event. It included a physical exam, a resting electrocardiogram (ECG), and an incremental maximal cardiopulmonary exercise test (CPET) on an electronically braked cycle ergometer. We used the French Cycling Federation incremental protocol with warm-up stage (100W/5min and 150W/1min) followed by a regular step load-increase (25W/min) until exhaustion.

### Cycling event description

Cyclists performed a long-lasting moderate-intensity stage cycling event that consisted of 21 stages and two rest days (Table 1).

**Table 1:** Characteristics of the multi-stage event.

Day	Stage	Distance (km)	Profile
D1	1^st^	188	Flat stage
D2	2^nd^	183	Flat stage
D3	3^rd^	223.5	Flat stage
D4	4^th^	237.5	Flat stage
D5	5^th^	216	Medium mountain stage
D6	6^th^	190.5	Flat stage
D7	7^th^	162	Medium mountain stage
D8	8^th^	184	High mountain stage
D9	9^th^	184.5	High mountain stage
D10	Rest		Rest Day
D11	10^th^	197	Flat stage
D12	11^th^	162.5	Flat stage
D13	12^th^	178	High mountain stage
D14	13^th^	37.5	Hilly stage
D15	14^th^	208.5	Flat stage
D16	15^th^	160	High mountain stage
D17	16^th^	209	Flat stage
D18	Rest		Rest Day
D19	17^th^	184.5	High mountain stage
D20	18^th^	17	Short high mountain stage
D21	19^th^	146	High mountain stage
D22	20^th^	146.5	High mountain stage
D23	21^th^	113	Flat stage

The event was carried out without spirit of competition, the goal was only to complete the 3529 km of the event to promote women cycling. Athletes traveled all the flat stages together but performed the mountain stages at her own pace. Due to the length of the stages, cyclists fed and hydrated continuously on the bike, according their needs. The individual fluid intake was also free during the recovery period between each stage.

### Echocardiography

All subjects underwent resting echocardiography using a Vivid Q (GE Vingmed Ultrasound AS, Horten, Norway) the day before the start of the race, on 5 time points chosen according to the characteristics of the stage (flat and mountainous) and when logistics allowed (Table 1). They were all performed post-stage, as close to exercise cessation as possible (between 30 and 180 min) on arrival at the hotel located at sea level. All data were stored on a workstation for offline analysis (EchoPAC BT12; GE Vingmed Ultrasound AS) by a single cardiologist blinded to the clinical data. For each measurement, at least two cardiac cycles were averaged. Left ventricle end-diastolic (LVED) and end-systolic (LVES) diameters and LVED wall thickness were measured in parasternal views [23]. LV volumes and ejection fraction were measured by the biplane methods; peak E- wave and A-wave velocities of the mitral inflow were measured using pulsed-wave Doppler. Tissue Doppler imaging was recorded at the level of septal and lateral mitral annulus, to obtain the average peak velocities during systole (s’) and early (e’) and late (a’) diastole. The E/e’ ratio was calculated to assess LV filling pressure. Right ventricular (RV) morphological parameters were assessed using RV end-diastolic basal (RVD1) and mid cavity diameter (RVD2), RVED and RVES areas [23].

Longitudinal myocardial deformations, based on the speckle-tracking approach, were evaluated from standard two-dimensional images (at frame rates of ≥60 sec^-1^). Global longitudinal strain (GLS) was the average of the 18 segmental strain values from the apical four-, three-, and two-chamber views [24]. Post-systolic shortening was not included in the global strain analysis. The time to maximal myocardial shortening, including post-systolic shortening if present, was measured from the electrocardiographic onset Q wave in the 18 LV segments. RV global longitudinal strain (RV GLS) was determined as the average of the 3 RV free wall segments, from an apical four-chamber image focused on the RV as recommended [25].

Left atrial (LA) and right atrial (RA) longitudinal strain was assessed on the six segments of apical four-chamber view as recommended [25]. The ECG-derived R-wave was taken as the first reference frame. Peak and pre-atrial contraction values of LA longitudinal strain [L-e-Max (reservoir function) and L-e-PA (pump function), respectively] were calculated by averaging the values observed in the LA segments. Difference between peak and pre-atrial contraction values of LA longitudinal strain was calculated [Le-p = L-e-Max—L-e-PA; (conduit function)]. The same analysis was done with the RA strain.

### Exercise load analysis

HR and GPS data were monitored continuously during each stage with a HR monitor (Polar V800, Kempele, Finland). Power data were collected using powermeter pedals (Powertap P1). We used mean HR (% of maximal HR) and mean normalized power output, (% of maximal aerobic power output) to calculate individual objective exercise daily load. The Borg CR 10 scale was used to determine the individual rate of perceived exertion (RPE), indicative of subjective load [26].

### Statistical analysis

A Shapiro-Wilk test was used to confirm the normal Gaussian distribution of the data. Quantitative data were expressed as mean ± standard deviation (SD). An analysis of variance for repeated measurements was used to analyze the evolution of the different echocardiographic parameter over time was used. A Bonferoni correction was applied. Significance was set at *p*<0.05. Statisticall analysis was perform using Statistica v.7.1.

## Results

### General and exercise related-data

Athletes’ mean age was 29.4 ± 2.7 years (Table 2).

**Table 2:** Individual characteristics of the subjects participating in the case study.

	Age	Weight	Height	BMI	Previous cycling experience	Training	$\dot{V}{\text{O}}_{2}$ max	Maximal Aerobic Power Output	Relative Maximal Aerobic Power Output
	(years)	(kg)	(cm)	(kg.m^-2^)	(years)	(km.week^-1^)	(ml.min.kg^-1^)	(watts)	(W.kg^-1^)
**Subject 1**	27	57.2	164	21.20	5	250	57.6	320	5.6
**Subject 2**	26	64.8	170	22.10	3	200	48.4	260	4.0
**Subject 3**	30	50.4	146	24.40	16	350	55.5	280	5.6
**Subject 4**	30	52.3	159	20.60	6	300	50.4	300	5.7
**Subject 5**	34	61.5	167	21.50	18	100	45.3	275	4.5
**Subject 6**	28	78.2	170	24.90	5	250	42.0	300	3.8
**Subject 7**	31	53.2	165	18.70	17	300	75.1	280	5.3
**Mean ± SD**	29.4 ± 2.7	59.6 ± 9.7	163.0 ± 8.4	21.9 ± 2.1	10.0 ± 6.6	250.0 ± 81.6	53.5 ± 11.0	287.9 ± 20.0	4.9 ± 0.8

$\dot{V}{\text{O}}_{2}$ max: maximum oxygen uptake.

They had an average 10.0 ± 6.6 years’ experience in cycling, and reported an average weekly training of 250 km. Table 2 shows their anthropometrical and physiological characteristics and the results of the baseline CPET. Baseline maximum oxygen uptake and maximal aerobic power output were 53.5±11.0 mL.min.kg^-1^ and 287.9 ± 20.0 W, respectively.

All participants successfully completed the whole circuit (3529 km with 21 stages and 2 days of rest) within approximately 9236 minutes (154 hours), with a mean stage duration of 440 minutes (7 hours and 20 minutes).

The average percentage of maximal HR sustained during each stage was between 58 and 71%, with a mean of 65%. The average percentage of maximal aerobic power output was between 38 and 52% with a mean of 45% (normalized power). Lastly, the average RPE was between 1 and 7 with a mean of 4 (Table 3).

**Table 3:** Stage-associated exercise load.

Stage	Duration	Mean normalized power output	% Wmax	Mean HR	% HRmax	Maximum HR	RPE
	(min)	(Watt)		(bpm)		(bpm)	(n.u)
1^st^	397	119 ± 10	40 ± 5	128 ± 8	68 ± 5	172 ± 4	4 ± 2
2^nd^	472	121 ± 9	41 ± 5	124 ± 3	63 ± 5	181 ± 4	4 ± 2
3^rd^	554	120 ± 10	41 ± 3	118 ± 5	60 ± 4	164 ± 6	6 ± 2
4^th^	532	122 ± 12	42 ± 5	122 ± 7	65 ± 5	162 ± 3	5 ± 3
5^th^	569	134 ± 9	45 ± 4	126 ± 6	67 ± 4	174 ± 10	7 ± 2
6^th^	456	119 ± 7	41 ± 3	122 ± 9	65 ± 6	168 ± 5	4 ± 2
7^th^	421	134 ± 12	46 ± 6	129 ± 5	69 ± 3	165 ± 9	4 ± 2
8^th^	561	137 ± 9	48 ± 6	130 ± 7	69 ± 4	163 ± 8	5 ± 2
9^th^	586	131 ± 8	44 ± 4	127 ± 7	68 ± 3	181 ± 31	7 ± 2
Rest							
10^th^	470	131 ± 12	44 ± 4	123 ± 3	66 ± 2	181 ± 31	5 ± 3
11^th^	386	127 ± 15	44 ± 6	114 ± 7	61 ± 4	181 ± 32	2 ± 1
12^th^	478	135 ± 12	47 ± 5	117 ± 5	64 ± 5	179 ± 39	4 ± 1
13^th^	105	150 ± 15	52 ± 6	123 ± 6	66 ± 4	184 ± 37	2 ± 1
14^th^	505	134 ± 15	46 ± 6	117 ± 5	62 ± 4	166 ± 8	4 ± 2
15^th^	485	149 ± 10	52 ± 5	129 ± 6	69 ± 3	173 ± 20	5 ± 1
16^th^	529	114 ± 14	39 ± 5	109 ± 6	59 ± 4	153 ± 11	4 ± 1
Rest							
17^th^	491	142 ± 11	49 ± 5	130 ± 7	69 ± 3	176 ± 26	5 ± 2
18^th^	84	144 ± 8	48 ± 5	133 ± 8	71 ± 4	175 ± 20	2 ± 0
19^th^	517	143 ± 9	49 ± 5	127 ± 6	68 ± 2	165 ± 9	5 ± 1
20^th^	461	143 ± 16	48 ± 5	120 ± 4	64 ± 4	164 ± 10	4 ± 1
21^th^	179	106 ± 10	38 ± 3	115 ± 6	58 ± 3	156 ± 4	1 ± 1

Wmax. Maximal Aerobic Power Output; HR. Heart Rate; RPE. rate of perceived exertion

Data are presented as mean ± SD

Mean body weight was significantly reduced after 21 days of cycling (59.6 ± 9.7 *vs*. 58.2 ± 9.4 kg, *p = 0*.*02)*. From the CPET performed 48 hours after the end of the event, we observed an increase of maximal aerobic power output (287.9 ± 20.0 to 328.3 ± 27.1 W, *p = 0*.*03*; 4.9 ± 0.8 to 5.6 ± 0.7 W/kg, *p = 0*.*01*) without any change in maximal oxygen uptake (53.5 ± 11.0 vs. 52.9 ± 7.5 mL.min.kg^-1^, *p = 0*.*9*).

### Cardiac morphology

Resting HR measured at the time of echocardiography did not vary during the event (Table 4).

**Table 4:** Rest cardiac dimensions measured by echocardiography.

	Baseline	D3	D7	D12	D13	D23
**Resting heart rate**	55.7 ± 6.8	68.2 ± 11.6	68.0 ± 8.3	56.7 ± 6.8	55.5 ± 6.2	63.3 ± 8.3
**LV Morphology**						
LV ED D (mm)	48.9 ± 4.0	49.6 ± 4.2	47.4 ± 3.5	46.7 ± 2.7	47.7 ± 2.4	47.6 ± 1.6
LV ED D i (mm.m^-2^)	30.2 ± 3.7	30.5 ± 4.4	29.4 ± 3.8	28.9 ± 3.3	29.5 ± 3.0	29.4 ± 2.5
LV ES D (mm)	28.9 ± 4.0	30.4 ± 1.4	29.1 ± 3.0	28.8 ± 1.1	29.8 ± 2.3	29.1 ± 0.9
LV ES D i (mm.m^-2^)	18.0 ± 3.8	18.7 ± 1.8	18.1 ± 3.0	17.7 ± 1.3	18.4 ± 2.6	18.0 ± 1.8
LV ED V (mL.m^-2^)	97.0 ± 22.4	96.3 ± 15.0	89.6 ± 19.5	90.5 ± 10.2	90.1 ± 15.3	79.5 ± 17.1
LV ED V i (mL.m^-2^)	59.4 ± 11.3	59.0 ± 9.0	54.4 ± 7.3	55.5 ± 4.3	55.1 ± 6.5	48.6 ± 7.3
LV ES V (mL.m^-2^)	29.7 ± 8.6	32.1 ± 6.6	26.7 ± 4.4	30.2 ± 4.2	30.7 ± 9.6	26.9 ± 7.5
LV ES V i (mL.m^-2^)	18.2 ± 5.0	19.8 ± 4.6	16.3 ± 1.7	18.5 ± 2.1	18.8 ± 4.9	16.5 ± 4.0
Stroke volume (mL)	67.3 ± 15.6	64.2 ± 9.2	62.9 ± 16.7	60.3 ± 8.1	59.3 ± 10.1	52.5 ± 11.4
IVS ED D (mm)	8.4 ± 0.7	8.2 ± 0.8	7.9 ± 0.8	7.6 ± 0.3	7.8 ± 0.7	7.6 ± 0.8
IVS ED D i (mm.m^-2^)	5.2 ± 0.4	5.0 ± 0.7	4.9 ± 0.7	4.7 ± 0.5	4.8 ± 0.6	4.7 ± 0.7
LV PW ED (mm)	8.2 ± 0.8	8.2 ± 0.7	8.1 ± 0.7	7.9 ± 0.7	7.3 ± 1.0	7.5 ± 0.8
LV PW ED i (mm.m^-2^)	5.0 ± 0.5	5.0 ± 0.7	5.0 ± 0.4	4.9 ± 0.7	4.5 ± 0.7	4.6 ± 0.3
**RV Morphology**						
RV EDA (cm^-2^)	14.9 ± 2.2	15.7 ± 2.1	15.4 ± 1.8	15.5 ± 4.9	17.7 ± 3.4	15.7 ± 2.6
RV EDA i (cm^-2^.m^-2^)	9.1 ± 1.2	9.7 ± 1.8	9.5 ± 1.3	9.6 ± 3.3	11.0 ± 2.8	9.6 ± 1.5
RV ESA (cm^-2^)	8.1 ± 1.3	8.5 ± 1.1	8.1 ± 1.5	8.4 ± 2.6	9.6 ± 2.4	8.0 ± 2.2
RV ESA i (cm^-2^.m^-2^)	5.0 ± 0.7	5.2 ± 1.0	5.0 ± 1.1	5.2 ± 1.8	6.0±1.7	4.9 ± 1.3
RV D1 (mm)	34.1 ± 3.5	39.5 ± 4.9	33.6 ± 2.9	33.7 ± 4.4	36.0 ± 2.9	37.0 ± 4.1
RV D1 i (mm.m^-2^)	21.1 ± 2.7	24.4 ± 4.6	20.7 ± 2.1	20.9 ± 4.0	22.4 ± 3.4	22.9 ± 3.4
RV D2 (mm)	27.9 ± 2.2	28.9 ± 1.8	30.1 ± 3.6	30.2 ± 3.5	30.6 ± 2.0	31.0 ± 4.0
RV D2 i (mm.m^-2^)	17.3 ± 2.3	17.8 ± 2.5	18.6 ± 2.9	18.7 ± 3.3	18.9 ± 2.1	19.0 ± 2.0
**LA Morphology**						
LA V (mL)	42.5 ± 12.1	49.1 ± 9.5	49.8 ± 9.5	40.5 ± 10.4	46.0 ± 5.7	40.0 ± 12.4
LA V i (mL.m^-2^)	25.9 ± 6.3	30.4 ± 7.9	30.9 ± 7.5	24.8 ± 5.9	28.2 ± 4.3	24.3 ± 6.3
**RA Morphology**						
RA V (mL)	33.7 ± 10.0	39.7 ± 6.8	36.9 ± 13.8	39.2 ± 7.2	39.8 ± 4.7	33.1 ± 6.3
RA V i (mL.m^-2^)	20.7 ± 5.9	24.5 ± 5.4	22.2 ± 6.6	24.1 ± 4.2	24.5 ± 2.9	20.3 ± 3.4

S. surface; D. diameter. V. volume; RA. right atria; LA. left atria; RV. right ventricle; LV. left ventricle; ED. end-diastolic; ES. end-systolic; Ao. aorte; IVS. intra ventricular septum; PW. posterior wall; RV D1. basal RV linear dimension; RV D2. mid-cavity RV linear dimension; i. indexed for body surface area

Data are presented as mean ± SD. Statistical differences with the baseline: * (*p* < 0.05). ** (*p* < 0.01). *** (*p* < 0.001).

Resting cardiac dimensions did not significantly change whatever the cavity analyzed; indeed none of the volumes, diameters or surfaces at D3, D7, D12, D13 or D23 time point was significantly different from baseline.

### Cardiac function

LV systolic and diastolic function did not significantly change during the 23 days of cycling (Table 5).

**Table 5:** Rest cardiac function parameters measured by echocardiography.

	Baseline	D3	D7	D12	D13	D23
**LV systolic function**						
EF (%)	69.5 ± 4.1	66.9 ± 3.1	69.6 ± 4.5	66.5 ± 3.6	66.2 ± 6.8	66.2 ± 5.5
S’ (cm.s^-1^)	9.6 ± 1.4	10.0 ± 2.1	10.8 ± 0.6	10.1 ± 1.1	10.7 ± 0.7	11.4 ± 1.7
Global L Strain (%)	-21.6 ± 2.4	-17.7 ± 5.3	-22.2 ± 2.1	-21.8 ± 2.6	-21.0 ± 2.0	-19.3 ± 2.1
**LV diastolic function**						
E (cm.s^-1^)	91.3 ± 11.5	85.4 ± 4.8	106.4 ± 13.7	88.7 ± 11.5	87.0 ± 16.3	84.7 ± 11.1
A (cm.s^-1^)	41.3 ± 12.0	45.8 ± 5.2	48.9 ± 8.5	46.4 ± 8.1	42.4 ± 6.9	49.1 ± 10.1
E/A	2.4 ± 0.8	1.9 ± 0.3	2.2 ± 0.4	2.0 ± 0.5	2.1 ± 0.6	1.8 ± 0.5
E/e’	5.7 ± 1.3	5.9 ± 0.7	6.0 ± 1.1	5.8 ± 0.5	5.5 ± 0.9	5.3 ± 0.8
e’ (cm.s^-1^)	16.7 ± 2.9	14.7 ± 1.6	18.1 ± 2.9	15.4 ± 2.3	15.8 ± 2.2	16.3 ± 2.7
a’ (cm.s^-1^)	7.1 ± 2.7	6.8 ± 1.1	14.1 ± 10.5	6.9 ± 1.3	7.1 ± 1.2	8.6 ± 1.2
**RV systolic function**						
TAPSE (mm)	23.9 ± 4.4	26.4 ± 3.4	27.4 ± 4.3	23.8 ± 4.7	25.0 ± 4.8	21.6 ± 3.4
FAC (%)	45.3 ± 7.1	45.8 ± 1.9	47.6 ± 8.7	43.3 ± 6.5	43.1 ± 8.2	48.8 ± 8.0
S’ (cm.s^-1^)	12.7 ± 1.7	14.4 ± 1.5	17.0 ± 2.0	13.9 ± 2.2	15.0 ± 2.3	14.7 ± 3.5
Global L Strain (%)	-25.6 ± 2.2	-28.0 ± 2.8	-27.0 ± 5.4	-25.2 ± 2.4	-25.3 ± 5.5	-25.9 ± 3.1
**RA function**						
Reservoir (%)	47.8 ± 15.5	35.0 ± 13.8	39.0 ± 9.7	43.6 ± 14.8	40.9 ± 10.2	34.5 ± 13.3
Conduit (%)	37.6 ± 10.5	23.0 ± 11.2	29.6 ± 9.8	32.3 ± 11.6	30.7 ± 9.4	22.7 ± 9.9
Pump (%)	10.2 ± 5.9	12.0 ± 3.6	9.4 ± 1.5	11.3 ± 6.1	10.2 ± 1.6	11.8 ± 5.7
**LA function**						
Reservoir (%)	44.5 ± 9.9	37.6 ± 7.5	45.6 ± 7.2	53.0 ± 16.2	57.4 ± 20.0	43.3 ± 5.7
Conduit (%)	31.9 ± 8.5	24.9 ± 6.4	32.4 ± 7.7	39.6 ± 13.8	45.1 ± 20.7	29.2 ± 6.6
Pump (%)	12.6 ± 2.4	12.7 ± 1.2	13.2 ± 2.1	13.4 ± 3.0	12.2 ± 4.0	14.1 ± 4.3

TAPSE. tricuspid annular plane systolic excursion; EF. ejection fraction; SF. shortening fraction; L. longitudinal; S E et A. Peak S. E and A-wave velocities of the mitral inflow using pulsed-wave Doppler; LV S’ E’ et A’ peak velocities during systole (S’) and early (E’) and late (A’) diastole using tissue doppler imaging at mitral annulus (tricuspide annulus for RV).

Data are presented as mean ± SD. Statistical differences with the baseline: * (*p* < 0.05). ** (*p* < 0.01). *** (*p* < 0.001).

None of the EF, tissue doppler peak velocity (s’) or GLS values was different from baseline. RV systolic function evaluated by TAPSE (Tricuspid annular plane systolic excursion), FAC (Fractional area change), tissue Doppler or strain, was unchanged. Concerning atria, we also noted no alteration of LA and RA function. Reservoir, conduit and pump functions were not modified all along the event.

## Discussion

The aim of this case study was to investigate the impact of a long-lasting and moderate-intensity stage cycling event on cardiac function of 7 well-trained young female cyclists. No significant modification was observed all along the event, whatever the cavity studied. Thus, the repeated and prolonged moderate endurance exercise performed by our population seems insufficiently stressful to impair cardiac function.

### Effects of cycling event on echocardiographic ventricular parameters

The moderate-intensity cycling event performed did not negatively alter the systolic bi-ventricular function and the diastolic LV function of the athletes studied. At a first glance, these results tend to disagree with most publications on this topic. Indeed, previous data reported a transient decrease of ventricular systolic function after prolonged multistage exercise [7, 10, 16–18]. The systolic dysfunction severity seemed to be positively related to the characteristics of the endurance exercise carried out such as its intensity and duration. Indeed, the more intense, prolonged and repeated exercise, the greater the alteration of ejection fraction [7, 10, 16–18]. In our study, athletes completed a very long duration cycling event: 23 days, 21 stages, 3529 km, 154 hours with a with a mean stage duration of 7 hours and 20 minutes; longer than in most previous studies [7, 10, 16–18]. For example, in these, the event included between 2 and 20 stages. But, with respect to the percentage of maximal heart rate (58–71%) and maximal aerobic power output (38–52%) sustained during each stage, the intensity of exercise was moderate and less intense than in most previous studies [7, 10, 16–18]. For example, in these, the percentage of maximal heart rate sustained during each stage was between 55 and 85. The shorter the event (number of stage), the higher the sustained intensity. Thus, the lower exercise intensity in our study could explain, at least in part, the absence of signs of systolic bi-ventricular and LV diastolic dysfunction. Indeed, the prolonged moderate aerobic exercise maintained during all the event by the cyclists may allow the cardiovascular system to adapt to prolonged moderate stress, thereby minimizing the probability of adverse effects on cardiac function [16]. This could also explain the lack of $\dot{\text{V}}{\text{O}}_{2}$ max improvement observed in athletes [27]. The observed increase of the mechanical performance (*i*.*e* maximal power output) at the end of the event could be explained by a better pedaling efficiency [28] although we cannot measure it.

Sex could also play a role in the lack of systolic bi-ventricular and LV diastolic dysfunction related to exercise observed in this study. Indeed, only studies including exclusively male athletes have reported a decrease in ventricular function [7, 10, 16–18]. Therefore, the absence of echocardiographic cardiac fatigue signs we observed could be due to a better cardiovascular tolerance of female athletes to prolonged moderate endurance exercise. Although the cardiovascular response of female athletes to long duration exercise are poorly documented, Danielsson et al. (2017) reported lower myocardial damage assessed by biomarkers, in female than in male triathletes after an ironman-distance triathlon [29].

### Effects of cycling event on echocardiographic atrial parameters

We observed no alteration in right and left RA atrial function all along the event. Reservoir, conduit and pump function were not modified whatever the cavity considered. This is not consistent with a previous study, which demonstrated that right atrial reservoir function was the first to be affected by the increase in endurance exercise load [13, 30]. According to our previous study, the echocardiographic cascade of alterations indicative of exercice-induced cardiac fatigue seems to begin with an alteration of speckle tracking derived parameters of diastolic function. (i.e., atrial reservoir function) [13]. Thus, as explained for the ventricular function, the absence of atrial function alteration can be explained by the fact that the cardiovascular workload induces by the event it not enough to induce the beginnings of exercise-induced cardiac fatigue.

### Limitations of the study

The major limitation of this study is the small number of athletes, which inevitably limits the scope of the study. Indeed, with our small sample, we cannot deny a possible type 2 error that is a non-rejection of the null hypothesis when it would be rejected with a larger sample. However, it must be taken into account the great technical difficulty of studying more athletes during such a long stage endurance event. These technical difficulties associated with the heavy and specific daily logistic (time constraints) also explain why we were not able to perform day-to-day assessments of cardiac function during the whole duration cycling event. Similarly, the immediate return to real life of the athletes at the end of the event (each in her city/country) did not allow following them up few hours and/or few days later. Finally, due to the long time of the study (23 days), it was impossible for us to get support from a single cardiologist. This constitue a bias due to possible inter-operator variability.

## Conclusions and perspectives

In the context of our study, the long-lasting moderate-intensity stage cycling event was not associated with cardiac function alteration. Our results are rather reassuring for medical community and athletes given their increase participation in ultra-endurance events. Nevertheless, we must be careful in interpreting them due to the limits of an underpowered study. Further analysis should be performed on a much larger sample to complete this study case.
